# Dietary inflammatory index and gestational diabetes mellitus in a multicenter cross sectional study in Suzhou

**DOI:** 10.3389/fimmu.2026.1909211

**Published:** 2026-10-05

**Authors:** Caifeng Wang, Yuanyuan Yang, Yueming Zhang, Xinzhi Wang, Yueyue Sun, Mindan Xu, Li Pu

**Affiliations:** 1 Department of Obstetrics and Gynecology, The Fourth Affiliated Hospital of Soochow University, Suzhou, China; 2 Department of Emergency, The Second Affiliated Hospital of Soochow University, Suzhou, China; 3 Department of Obstetrics and Gynecology, The First Affiliated Hospital of Soochow University, Suzhou, China

**Keywords:** China, cross-sectional study, dietary inflammatory index, dietary pattern, gestational diabetes mellitus, inflammation, pregnancy

## Abstract

**Background:**

Evidence linking dietary inflammatory potential with gestational diabetes mellitus (GDM) has been inconsistent. We examined this association in a multicenter cross-sectional study.

**Methods:**

Women attending three tertiary hospitals in Suzhou, China, from February to November 2025 completed a food-frequency questionnaire at 24 to 28 gestational weeks, recalling the preceding three months. A 26-parameter Dietary Inflammatory Index (DII) was calculated, and GDM was defined from 75-g oral glucose tolerance test (OGTT) values. Logistic regression adjusted for demographic, socioeconomic, obstetric, behavioral, and hospital covariates.

**Results:**

The primary analysis included 1,071 women after removal of 23 exact duplicate records; 225 (21.0%) had GDM. Mean age was 29.28 years (SD 4.17), prepregnancy body mass index was 22.42 kg/m² (SD 3.28), and DII was 0.71 (SD 1.13). Each one-SD higher DII was associated with higher adjusted GDM odds (OR 1.26, 95% CI 1.06 to 1.51; P = 0.011). The adjusted quartile trend was not significant (P = 0.162), and there was no evidence of nonlinearity (P = 0.888). Among 803 women completing the questionnaire one to three days before OGTT, the estimate was attenuated and imprecise (OR 1.20, 95% CI 0.97 to 1.48).

**Conclusion:**

Higher continuous DII was associated with GDM, but quartile and sensitivity analyses limited the consistency of the findings. Overlapping exposure and outcome windows and measurement limitations precluded causal or predictive interpretation.

## 1 Introduction

Gestational diabetes mellitus (GDM) is an important complication of pregnancy in China, although its reported prevalence depends strongly on diagnostic criteria, region, and study period. A systematic review of studies conducted from 1990 to 2024 estimated a pooled prevalence of 15.6% in the post-2010 subgroup using International Association of Diabetes and Pregnancy Study Groups (IADPSG) criteria. This estimate described heterogeneous study populations rather than a uniform national rate (1). GDM develops when pancreatic beta-cell compensation is insufficient for the insulin resistance of pregnancy; inflammatory processes may contribute to this metabolic imbalance (2).

Dietary patterns offer one way to investigate potentially relevant exposures without treating nutrients as independent of the foods in which they occur. Prospective studies in the United States and Australia have associated greater adherence to healthier prepregnancy dietary patterns with lower GDM incidence, but such observational findings do not identify inflammation as the mediating pathway (3, 4). The literature-derived Dietary Inflammatory Index (DII) was developed to characterize dietary inflammatory potential using 45 food and nutrient parameters, inflammatory effect scores derived from published research, and international reference intakes (5). Associations with C-reactive protein in nonpregnant adults provided construct-validation evidence, but they did not establish the validity of every shortened DII implementation in pregnancy (6).

Evidence linking DII to GDM has been inconsistent. The Tongji Maternal and Child Health Cohort reported a positive association in 2,639 Chinese women, whereas Project Viva and an Iranian prospective cohort reported inverse and null findings, respectively (7–9). Reviews have also differed: a 2023 systematic review concluded that the observational evidence did not support an overall association, while a later meta-analysis reported a modest positive pooled association with substantial heterogeneity (10, 11). Differences in exposure timing, dietary instruments, score construction, diagnostic criteria, and adjustment may contribute to the disagreement.

Chinese evidence is therefore not absent. In addition to the Tongji DII study, a larger Hefei cohort examined empirically derived inflammatory and insulinemic dietary patterns (12). Empirical food-pattern indices and the literature-derived DII represent related but distinct constructs (13). The present study adds a geographically specific assessment of a 26-parameter DII measured near routine GDM testing across three tertiary hospitals, including continuous, categorical, and nonlinear analyses and an explicit assessment of questionnaire timing. It does not provide stronger temporal evidence than a prospective cohort. We aimed to estimate the cross-sectional association between DII and GDM, with secondary analyses of quartile patterns, functional form, sensitivity to analytical decisions, and exploratory modification by prepregnancy body mass index (BMI) and parity.

## 2 Materials and methods

### 2.1 Study design and participants

This multicenter cross-sectional study included pregnant women receiving routine antenatal care at the Fourth, Second, and First Affiliated Hospitals of Soochow University in Suzhou, China, from February 21 to November 11, 2025. Eligible women were aged 18 to 45 years, had a singleton pregnancy, and attended at 24 to 28 gestational weeks. Exclusion criteria included preexisting type 1 or type 2 diabetes, multiple gestation, recent use of systemic corticosteroids or other medication affecting glucose metabolism, participation in another clinical trial, and more than 10% missing FFQ items. Reporting followed the nutritional epidemiology principles of STROBE-nut (14).

The study used the available eligible records from the recruitment period. No dated sample-size calculation or analysis plan established before outcome analysis was available; consequently, a retrospectively reconstructed calculation was not presented as prospective justification. Aggregate recruitment information recorded 1,250 invitations and 1,094 complete eligible records, but individual reasons for the difference of 156 were unavailable. The total number of potentially eligible antenatal attendees and the mechanism for selecting women for invitation were not documented, so representativeness and an eligible-population participation rate could not be established.

We identified 23 pairs of records matching exactly across the supplied analytical fields and retained one record from each pair for the primary analysis, leaving 1,071 records. Source-level identifiers were unavailable for adjudicating whether each pair represented duplicate entry of one participant. An analysis retaining all 1,094 records assessed the sensitivity of the findings to this decision. **Figure 1** summarizes recruitment and analytical populations.

**Figure 1:**
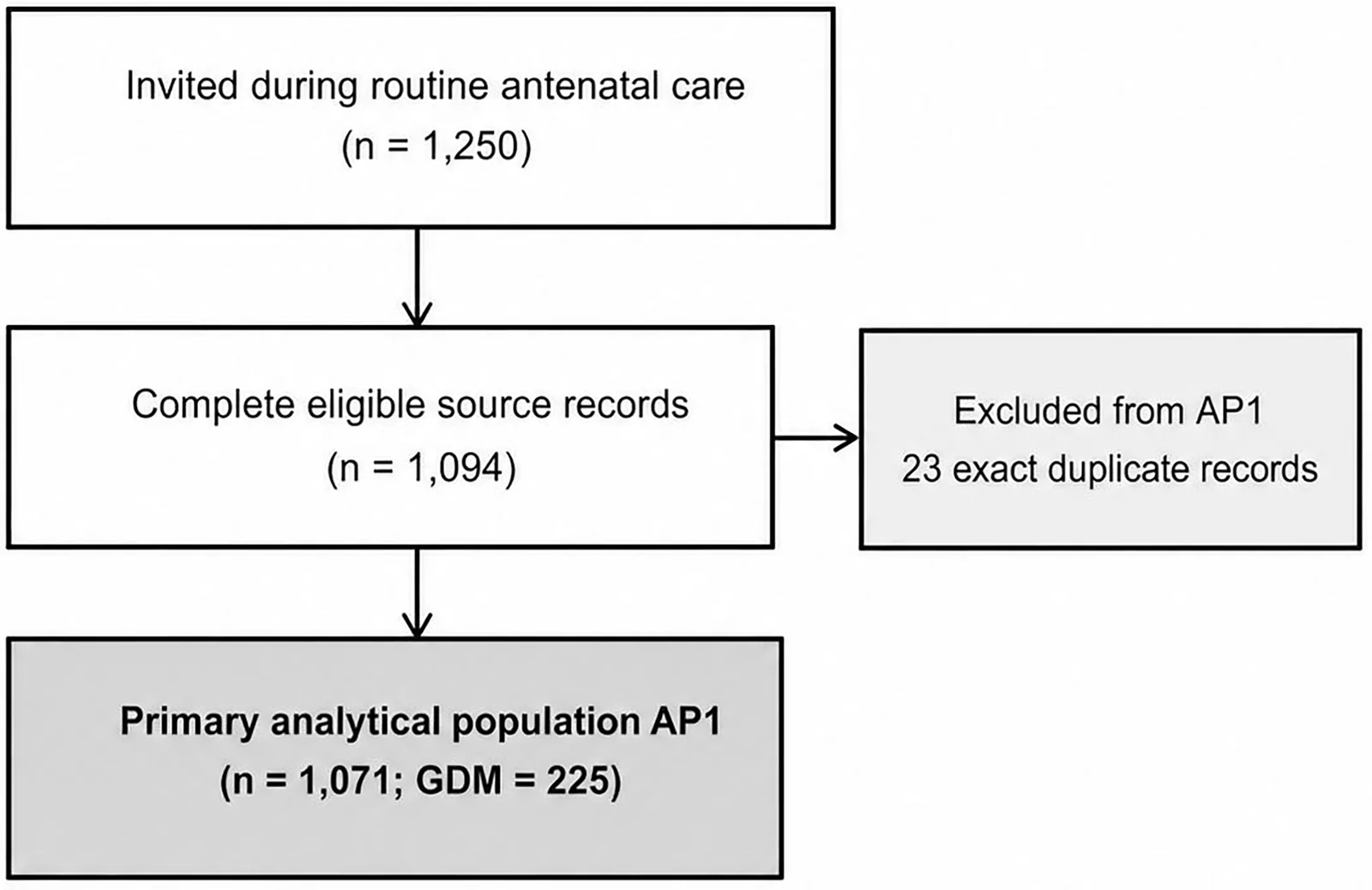
Participant flow. Invitation and eligibility totals were aggregate study counts; individual reasons for the difference of 156 were unavailable. The primary analysis removed one record from each exact duplicate pair without source-identity adjudication. AP, analytical population; GDM, gestational diabetes mellitus.

### 2.2 Dietary assessment and timing

Dietary intake during the preceding three months was assessed with a 103-item semiquantitative food-frequency questionnaire (FFQ). Responses used nine intake-frequency categories and three portion-size categories. The study instrument provided standardized frequency and portion-size coding rules. However, validation of the exact 103-item instrument and its equivalence to an independently validated questionnaire were not established. Daily food and nutrient intakes recorded in the master dataset were used for analysis. The underlying food-composition coefficient matrix and executable FFQ-to-nutrient conversion code were unavailable, which prevented independent reconstruction of nutrient intakes from individual FFQ responses.

Questionnaires were dated on the same day as the oral glucose tolerance test (OGTT) for 268 women, one day before for 283, two days before for 266, and three days before for 254. Prior disclosure of glucose results, clinical suspicion of hyperglycemia, and the timing of dietary counseling were not recorded. We therefore analyzed the 803 women whose questionnaire preceded the OGTT by one to three days as a timing-restricted sensitivity population. The preceding three-month dietary recall period still overlapped the development of GDM, even when questionnaire completion preceded testing.

### 2.3 Dietary inflammatory index calculation

We calculated the DII from the recorded nutrient intakes using published parameter-specific global means, standard deviations, and inflammatory effect scores (5). Each intake was standardized against its corresponding global reference distribution. The implemented transformation was 2Φ(z) − 1, where Φ denoted the standard normal cumulative distribution function. This centered percentile was multiplied by the inflammatory effect score, and contributions were summed. Higher DII values represented greater estimated proinflammatory dietary potential.

Twenty-six parameters had compatible intake units and reference constants: energy, carbohydrate, protein, total fat, fiber, cholesterol, saturated fat, monounsaturated fat, polyunsaturated fat, omega-3 and omega-6 fatty acids, beta-carotene, vitamins C, D, and E, thiamin, riboflavin, niacin, vitamins B6 and B12, folate, iron, magnesium, zinc, selenium, and alcohol. Calcium was not included because it was not a parameter in the original 45-component index. Vitamin A was excluded because the dataset used retinol activity equivalents, whereas the published reference used retinol equivalents; a defensible participant-level conversion was unavailable. The full list of included parameters, units, reference constants, and inflammatory effect scores appears in **Supplementary Table S1**.

The resulting score was an ordinary 26-parameter DII, including energy as one component. It was not the energy-adjusted DII (E-DII), which required a different energy-adjusted reference database (15). We standardized the continuous score using the primary analytical sample standard deviation and used empirical quartile cut points of 0.0189, 0.8643, and 1.5285. The same standard deviation and cut points were retained in sensitivity analyses.

### 2.4 GDM ascertainment

GDM status was derived from recorded routine clinical 75-g OGTT glucose values obtained at 24 to 28 gestational weeks. Classification followed IADPSG thresholds: fasting plasma glucose of at least 5.1 mmol/L, one-hour glucose of at least 10.0 mmol/L, or two-hour glucose of at least 8.5 mmol/L, with one abnormal value sufficient for GDM (16). The recalculated classification agreed with the stored binary GDM variable. Fasting glucose of at least 7.0 mmol/L or two-hour glucose of at least 11.1 mmol/L was additionally checked to distinguish overt diabetes first recognized in pregnancy.

The analysis used clinical glucose results rather than assays of stored research specimens. Detailed specimen-processing times, storage histories, analyzer calibration records, and interlaboratory quality-control records were unavailable for independent verification. Applying common diagnostic thresholds did not establish identical preanalytical handling across hospitals.

### 2.5 Covariates

The dataset included maternal age, prepregnancy BMI, education, monthly household income, parity, gestational week, physical activity, smoking, secondhand-smoke exposure, family history of type 2 diabetes, previous GDM, and hospital. Prepregnancy BMI was calculated from prepregnancy weight and height. Gestational weight gain was calculated as the difference between current and prepregnancy weight. Physical activity was recorded with a study-adapted self-report form covering the preceding three months and summarized as metabolic-equivalent minutes per week. It was entered continuously, not as an activity category. Validation of this exact simplified form in pregnancy was not established.

Covariates were selected for the reanalysis using the study design and clinical knowledge rather than univariable significance screening. This approach was consistent with principles of confounder selection based on plausible exposure and outcome determinants (17). Education, income, smoking, parity, secondhand smoke, family history, previous GDM, and hospital were modeled as categorical variables. Age, BMI, gestational week, and physical activity were modeled continuously. Hospital was included as a fixed effect; three hospitals were insufficient for reliable cluster-robust inference. Total energy and gestational weight gain were examined in separate sensitivity models. Supplement use and additional pregnancy complications were not included in the verified adjustment set, leaving potential residual confounding.

### 2.6 Statistical analysis

Continuous characteristics were summarized as mean and standard deviation (SD), and skewed dietary intakes as median and interquartile range. Categorical variables were presented as counts and percentages. Comparisons across DII quartiles used one-way analysis of variance for continuous participant characteristics, Kruskal-Wallis tests for dietary variables, and Pearson chi-square tests for categorical characteristics, except smoking, for which Fisher’s exact test was used because of sparse cells. No between-quartile test was applied to DII itself because the score defined the groups.

Binary logistic regression estimated odds ratios (ORs) and 95% confidence intervals (CIs). Model 1 was unadjusted, Model 2 adjusted for age and prepregnancy BMI, and the primary Model 3 additionally adjusted for the remaining covariates specified in Section 2.5. The primary contrast was a one-SD higher continuous DII. Quartile comparisons used Q1 as the reference, and the trend test entered quartile-specific median DII values as a continuous term. Restricted cubic splines used knots at the 10th, 50th, and 90th percentiles, with the median as the reference; overall and nonlinear associations were assessed with Wald tests.

Exploratory subgroup analyses considered prepregnancy BMI below 24 versus at least 24 kg/m² and nulliparous versus parous women. The stratification variable was omitted from the corresponding subgroup model. Product terms were tested in the full sample, and the two interaction P values were adjusted using the Benjamini-Hochberg procedure. No age-stratified analysis was conducted.

Sensitivity analyses retained exact duplicate records, excluded DII values below the first or above the 99th percentile, excluded women with a family history of type 2 diabetes, added total energy intake, added gestational weight gain, restricted questionnaire timing to one to three days before OGTT, omitted hospital, coded education and income ordinally, and combined former and current smokers. Energy adjustment was used to examine the influence of total intake, not to reconstruct E-DII. Different energy-adjustment formulations answer different nutritional epidemiology questions (18). Gestational weight gain was kept outside the primary model because it was measured contemporaneously and might partly follow the dietary exposure.

Missingness was evaluated for each primary-model variable; all were complete, and no imputation was performed (**Supplementary Table S2**). Diagnostics assessed convergence, sparse cells, variance inflation, influence, and continuous-covariate functional form. Analyses were conducted in R version 4.6.0. An independent implementation checked sample counts, GDM classification, DII calculation, and the primary regression result. Tests were two-sided, with P < 0.05 used as a descriptive significance threshold. Confidence intervals and consistency across analyses were considered alongside P values, and secondary and exploratory findings were not treated as confirmatory evidence (19).

## 3 Results

### 3.1 Study population and dietary characteristics

After removal of one record from each of 23 exact duplicate pairs, the primary analysis included 1,071 women, of whom 225 had GDM (21.0%; **Figure 1**). No woman met the specified fasting or two-hour thresholds for overt diabetes. Mean maternal age was 29.28 years (SD 4.17), and mean prepregnancy BMI was 22.42 kg/m² (SD 3.28).

DII had a mean of 0.71 (SD 1.13), a median of 0.86, and a range of −3.22 to 3.26 (**Figure 2**). Quartile sample sizes were 268, 268, 267, and 268. GDM occurred in 42 women in Q1 (15.7%), 50 in Q2 (18.7%), 62 in Q3 (23.2%), and 71 in Q4 (26.5%). **Table 1** presents participant characteristics and **Table 2** presents dietary intakes across quartiles. Differences in nutrient intake across score-defined groups were descriptive characteristics of the constructed exposure, not independent validation of its inflammatory properties.

**Figure 2:**
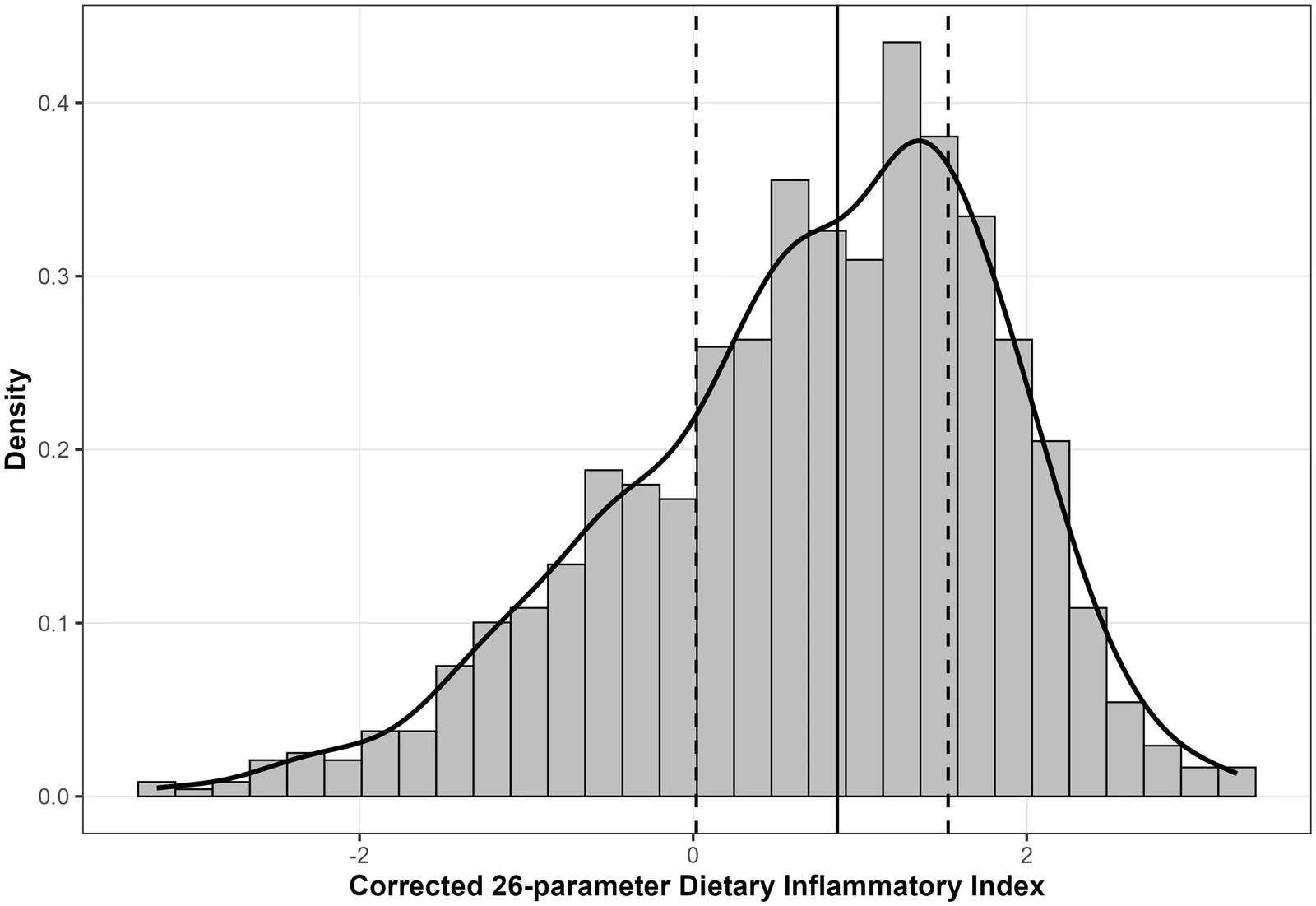
Distribution of the 26-parameter dietary inflammatory index in the primary analytical sample (n = 1,071). Vertical lines indicate quartile cut points of 0.0189, 0.8643, and 1.5285. DII, dietary inflammatory index.

**Table 1:** Participant characteristics by dietary inflammatory index quartile.

Characteristic	Overall	Q1	Q2	Q3	Q4	P value
Age, years	29.28 (4.17)	30.15 (3.85)	29.40 (3.99)	29.31 (4.26)	28.25 (4.35)	<0.001
Prepregnancy BMI, kg/m2	22.42 (3.28)	21.94 (2.90)	22.20 (3.17)	22.49 (3.31)	23.05 (3.63)	<0.001
Gestational week at enrollment	25.76 (1.31)	25.76 (1.30)	25.74 (1.25)	25.64 (1.36)	25.91 (1.32)	0.134
Physical activity, MET-min/week	1364.96 (713.02)	1326.44 (661.85)	1246.90 (592.73)	1456.17 (799.43)	1430.65 (763.68)	0.002
26-parameter DII	0.71 (1.13)	-0.85 (0.68)	0.47 (0.23)	1.22 (0.19)	2.00 (0.38)	Not tested
Education: Middle school or below	228 (21.3%)	31 (11.6%)	48 (17.9%)	65 (24.3%)	84 (31.3%)	<0.001
Education: High school/technical school	340 (31.7%)	66 (24.6%)	86 (32.1%)	99 (37.1%)	89 (33.2%)	
Education: College or above	503 (47.0%)	171 (63.8%)	134 (50.0%)	103 (38.6%)	95 (35.4%)	
Monthly household income: <5,000 RMB	257 (24.0%)	44 (16.4%)	54 (20.1%)	73 (27.3%)	86 (32.1%)	<0.001
Monthly household income: 5,000-10,000 RMB	475 (44.4%)	114 (42.5%)	115 (42.9%)	134 (50.2%)	112 (41.8%)	
Monthly household income: >10,000 RMB	339 (31.7%)	110 (41.0%)	99 (36.9%)	60 (22.5%)	70 (26.1%)	
Parity: Nulliparous	600 (56.0%)	167 (62.3%)	149 (55.6%)	142 (53.2%)	142 (53.0%)	0.102
Parity: Parous	471 (44.0%)	101 (37.7%)	119 (44.4%)	125 (46.8%)	126 (47.0%)	
Smoking: Never	1039 (97.0%)	258 (96.3%)	265 (98.9%)	262 (98.1%)	254 (94.8%)	0.049
Smoking: Former	22 (2.1%)	6 (2.2%)	1 (0.4%)	4 (1.5%)	11 (4.1%)	
Smoking: Current	10 (0.9%)	4 (1.5%)	2 (0.7%)	1 (0.4%)	3 (1.1%)	
Secondhand smoke exposure: No	680 (63.5%)	182 (67.9%)	159 (59.3%)	162 (60.7%)	177 (66.0%)	0.115
Secondhand smoke exposure: Yes	391 (36.5%)	86 (32.1%)	109 (40.7%)	105 (39.3%)	91 (34.0%)	
Family history of type 2 diabetes: No	816 (76.2%)	210 (78.4%)	207 (77.2%)	200 (74.9%)	199 (74.3%)	0.647
Family history of type 2 diabetes: Yes	255 (23.8%)	58 (21.6%)	61 (22.8%)	67 (25.1%)	69 (25.7%)	
Previous GDM: No	1030 (96.2%)	262 (97.8%)	259 (96.6%)	256 (95.9%)	253 (94.4%)	0.227
Previous GDM: Yes	41 (3.8%)	6 (2.2%)	9 (3.4%)	11 (4.1%)	15 (5.6%)	
Center: Fourth Affiliated Hospital	369 (34.5%)	88 (32.8%)	108 (40.3%)	87 (32.6%)	86 (32.1%)	0.437
Center: First Affiliated Hospital	349 (32.6%)	93 (34.7%)	77 (28.7%)	89 (33.3%)	90 (33.6%)	
Center: Second Affiliated Hospital	353 (33.0%)	87 (32.5%)	83 (31.0%)	91 (34.1%)	92 (34.3%)	
GDM	225 (21.0%)	42 (15.7%)	50 (18.7%)	62 (23.2%)	71 (26.5%)	0.011

Data are mean (standard deviation) or n (%). P values were obtained with one-way analysis of variance for continuous characteristics and Pearson chi-square tests for categorical variables, except smoking, which used Fisher’s exact test. No test was performed for DII because its quartiles defined the columns. BMI, body mass index; DII, Dietary Inflammatory Index; GDM, gestational diabetes mellitus; MET, metabolic equivalent.

**Table 2:** Dietary intake by dietary inflammatory index quartile.

Dietary variable	Overall	Q1	Q2	Q3	Q4	P value
Energy, kcal/day	2005.59 (1814.35, 2176.15)	2014.60 (1816.29, 2186.89)	1970.29 (1798.77, 2146.01)	2049.57 (1877.82, 2199.75)	1962.87 (1788.90, 2155.77)	0.003
Carbohydrate, g/day	204.23 (182.33, 229.53)	203.94 (181.66, 229.98)	198.78 (177.51, 226.49)	207.84 (185.37, 233.87)	206.19 (183.93, 227.47)	0.128
Protein, g/day	79.94 (70.70, 90.20)	86.39 (77.05, 96.21)	80.43 (70.80, 90.54)	78.96 (70.00, 87.80)	74.12 (66.43, 83.25)	<0.001
Total fat, g/day	95.86 (83.29, 108.89)	94.03 (81.82, 108.60)	95.19 (83.07, 108.88)	99.82 (88.90, 110.33)	93.37 (80.01, 107.55)	<0.001
Fiber, g/day	11.32 (9.46, 13.71)	14.91 (13.63, 17.02)	12.07 (11.10, 13.50)	10.43 (9.42, 11.43)	8.68 (7.73, 9.99)	<0.001
Refined grains, g/day	227.00 (180.10, 285.60)	181.05 (143.70, 222.27)	215.70 (176.88, 265.43)	251.60 (210.70, 307.10)	268.20 (223.08, 321.47)	<0.001
Whole grains, g/day	22.00 (10.00, 38.25)	39.05 (25.65, 56.00)	25.00 (13.15, 41.73)	16.30 (8.30, 29.95)	10.80 (5.22, 21.38)	<0.001
Vegetables, g/day	217.70 (159.50, 290.35)	290.40 (227.47, 375.43)	240.50 (191.47, 303.23)	198.30 (153.50, 251.45)	154.85 (119.40, 197.05)	<0.001
Fruit, g/day	180.90 (117.10, 261.05)	262.50 (188.13, 342.37)	189.30 (140.38, 256.20)	152.00 (107.55, 220.15)	116.95 (85.95, 181.90)	<0.001
Red and processed meat, g/day	80.60 (54.60, 113.20)	54.65 (37.10, 76.62)	73.15 (51.98, 100.82)	93.00 (69.95, 132.85)	104.80 (78.40, 137.12)	<0.001
Fish and seafood, g/day	41.10 (26.20, 65.90)	66.30 (44.00, 95.05)	48.60 (32.60, 67.85)	35.10 (25.20, 51.50)	24.65 (17.67, 40.10)	<0.001
Dairy, g/day	169.50 (111.20, 237.55)	214.55 (169.18, 290.20)	173.30 (118.40, 240.67)	149.10 (93.90, 222.95)	121.10 (80.35, 183.53)	<0.001
Nuts and seeds, g/day	12.30 (6.70, 22.90)	23.35 (14.55, 34.95)	15.00 (9.15, 23.50)	10.00 (5.30, 18.20)	6.45 (4.30, 9.70)	<0.001
Sugar-sweetened beverages, mL/day	83.20 (45.95, 137.05)	34.05 (17.00, 62.20)	69.35 (45.55, 104.60)	105.60 (73.00, 153.30)	136.85 (88.67, 201.65)	<0.001

Data are median (interquartile range). P values were obtained with Kruskal-Wallis tests. DII, Dietary Inflammatory Index.

### 3.2 Association between DII and GDM

Each one-SD higher DII was associated with higher adjusted odds of GDM in Model 3 (OR 1.26, 95% CI 1.06 to 1.51; P = 0.011; **Table 3**). Adjustment attenuated the unadjusted association. Relative to Q1, adjusted ORs were 1.04 (95% CI 0.65 to 1.68) for Q2, 1.28 (0.80 to 2.06) for Q3, and 1.35 (0.84 to 2.17) for Q4. The quartile-median trend was not statistically significant (P = 0.162).

**Table 3:** Association between dietary inflammatory index and gestational diabetes mellitus.

DII exposure	Model	Odds ratio (95% CI)	P value
Per 1-SD corrected DII	Model 1	1.38 (1.18 to 1.62)	<0.001
Per 1-SD corrected DII	Model 2	1.32 (1.12 to 1.56)	<0.001
Per 1-SD corrected DII	Model 3	1.26 (1.06 to 1.51)	0.011
Q2 vs Q1	Model 3	1.04 (0.65 to 1.68)	0.863
Q3 vs Q1	Model 3	1.28 (0.80 to 2.06)	0.303
Q4 vs Q1	Model 3	1.35 (0.84 to 2.17)	0.216
Linear trend across quartile medians	Model 3		0.162

Odds ratios and two-sided Wald P values were obtained with binary logistic regression. Model 1 was unadjusted. Model 2 adjusted for age and prepregnancy BMI. Model 3 additionally adjusted for education, income, parity, gestational week, physical activity, smoking, secondhand smoke, family history of type 2 diabetes, previous GDM, and center. CI, confidence interval; DII, Dietary Inflammatory Index; SD, standard deviation.

The restricted cubic spline showed an overall association (P = 0.035) but no evidence of departure from linearity (P for nonlinearity = 0.888; **Figure 3**). Confidence intervals widened toward the ends of the DII distribution. The analysis did not identify a threshold or a clinically useful cut point.

**Figure 3:**
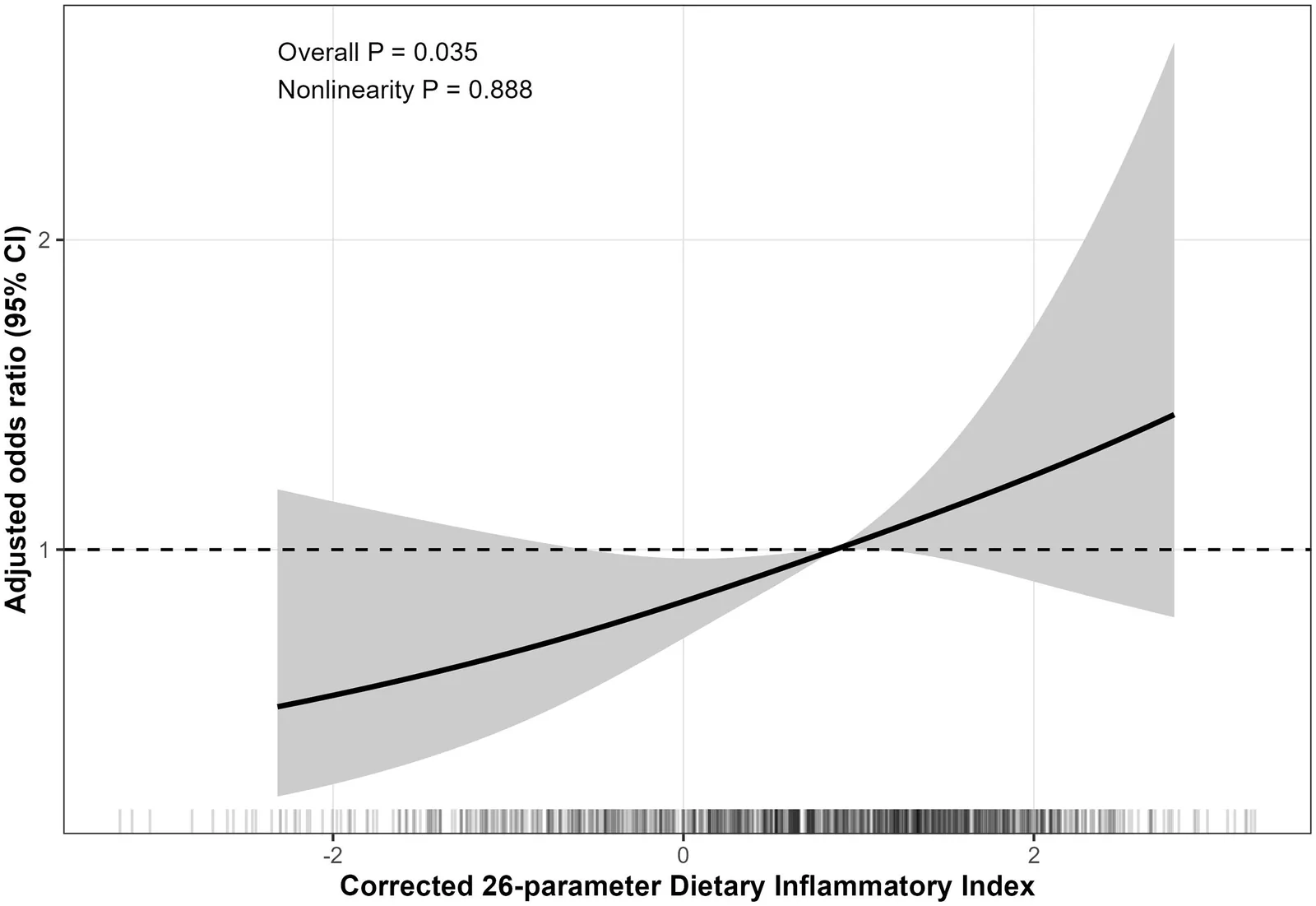
Restricted cubic spline for the adjusted association between the 26-parameter dietary inflammatory index and GDM. Knots were placed at the 10th, 50th, and 90th percentiles; the median was the reference. The line shows the adjusted odds ratio and the band its 95% confidence interval. Overall P = 0.035; nonlinear P = 0.888. Adjustment matched Model 3. DII, Dietary Inflammatory Index; GDM, gestational diabetes mellitus.

### 3.3 Hospital and exploratory subgroup analyses

Hospital-specific sample sizes, GDM prevalence, DII, age, and BMI are presented in **Table 4A**. In subgroup analyses, the adjusted OR per one-SD higher DII was 1.24 (95% CI 0.99 to 1.54) among women with prepregnancy BMI below 24 kg/m² and 1.35 (0.97 to 1.88) among women with BMI of at least 24 kg/m². The BMI interaction P value was 0.883. Corresponding estimates were 1.11 (0.86 to 1.43) among nulliparous women and 1.50 (1.15 to 1.94) among parous women. The parity interaction P value was 0.060 and the Benjamini-Hochberg-adjusted value was 0.120 (**Table 4B**). The subgroup results did not establish effect modification.

**Table 4A:** Center-specific participant and outcome summary.

Center	n	GDM	GDM prevalence	DII mean (SD)	Age mean (SD)	BMI mean (SD)
Fourth Affiliated Hospital	369	93	25.2%	0.68 (1.12)	29.20 (3.94)	22.51 (3.18)
First Affiliated Hospital	349	68	19.5%	0.73 (1.12)	29.23 (4.19)	22.45 (3.37)
Second Affiliated Hospital	353	64	18.1%	0.71 (1.15)	29.41 (4.38)	22.30 (3.32)

GDM prevalence is the number of GDM events divided by the center-specific sample. BMI, body mass index; DII, Dietary Inflammatory Index; GDM, gestational diabetes mellitus; SD, standard deviation.

**Table 4B:** Exploratory subgroup analyses..

Subgroup	n	GDM	Adjusted OR (95% CI)	P value	Interaction P	BH P
Prepregnancy BMI <24 kg/m2	728	136	1.24 (0.99 to 1.54)	0.056	0.883	0.883
Prepregnancy BMI >=24 kg/m2	343	89	1.35 (0.97 to 1.88)	0.075	0.883	0.883
Nulliparous	600	98	1.11 (0.86 to 1.43)	0.439	0.060	0.120
Parous	471	127	1.50 (1.15 to 1.94)	0.002	0.060	0.120

Odds ratios and two-sided Wald P values were obtained from Model 3 with the stratification variable omitted. Interaction P values came from product terms in the full sample; BH P values used Benjamini-Hochberg correction for two interaction tests. BMI, body mass index; BH, Benjamini-Hochberg; CI, confidence interval; DII, Dietary Inflammatory Index; GDM, gestational diabetes mellitus; OR, odds ratio.

### 3.4 Sensitivity analyses and diagnostics

Estimates remained similar when all source records were retained (OR 1.27, 95% CI 1.07 to 1.52), total energy was added (OR 1.28, 1.07 to 1.54), gestational weight gain was added (OR 1.27, 1.06 to 1.51), or hospital was omitted (OR 1.25, 1.05 to 1.49). Excluding women with a family history of type 2 diabetes, changing socioeconomic coding, and combining former and current smokers also produced positive estimates (**Table 5**).

**Table 5:** Sensitivity analyses.

Analysis	n	GDM	Adjusted OR (95% CI) per 1-SD DII	P value
Primary analysis	1071	225	1.26 (1.06 to 1.51)	0.011
Exact duplicates retained	1094	228	1.27 (1.07 to 1.52)	0.008
Exclude DII below 1st or above 99th percentile	1049	220	1.19 (0.99 to 1.44)	0.066
Exclude family history of type 2 diabetes	816	137	1.29 (1.04 to 1.61)	0.023
Additional total-energy adjustment	1071	225	1.28 (1.07 to 1.54)	0.007
Additional gestational-weight-gain adjustment	1071	225	1.27 (1.06 to 1.51)	0.010
Questionnaire 1–3 days before OGTT	803	168	1.20 (0.97 to 1.48)	0.092
Omit study center	1071	225	1.25 (1.05 to 1.49)	0.014
Ordinal education and income coding	1071	225	1.26 (1.05 to 1.51)	0.011
Collapse former and current smoking	1071	225	1.27 (1.06 to 1.52)	0.009

Odds ratios and two-sided Wald P values were obtained with binary logistic regression. Models used the primary Model 3 covariates except where the analysis description indicates a change. The family-history-exclusion model omitted family history. CI, confidence interval; DII, Dietary Inflammatory Index; GDM, gestational diabetes mellitus; OR, odds ratio; SD, standard deviation.

The estimate was attenuated after excluding the extreme 1% of DII values at each tail (OR 1.19, 95% CI 0.99 to 1.44; P = 0.066). Among the 803 women who completed the questionnaire one to three days before OGTT, 168 had GDM, and the adjusted OR was 1.20 (95% CI 0.97 to 1.48; P = 0.092). Both intervals included the null and overlapped the primary interval.

The primary model converged, and the maximum variance-inflation factor was 1.40. No GDM event occurred among the 10 current smokers, producing a sparse-cell concern for that covariate. Combining current and former smokers yielded a similar DII estimate (OR 1.27, 95% CI 1.06 to 1.52). No primary-model variable had missing data.

## 4 Discussion

### 4.1 Principal findings

In this three-hospital cross-sectional study, a one-SD higher 26-parameter DII was associated with 26% higher adjusted odds of GDM. The continuous estimate was positive, but the adjusted quartile comparisons were imprecise and the trend test was not significant. Neither the spline nor the subgroup analyses established a nonlinear threshold or effect modification. Estimates were also attenuated when extreme scores or same-day questionnaires were excluded. The findings support a modest contemporaneous association with material uncertainty, rather than a demonstrated causal effect or a validated clinical marker.

### 4.2 Comparison with previous studies

The positive continuous estimate is compatible in direction with the Tongji cohort, which reported an adjusted OR of 1.43 (95% CI 1.05 to 1.95) for the highest versus lowest DII tertile among 2,639 Chinese women (7). A Finnish study of 351 women with overweight or obesity reported an OR of 1.27 (1.08 to 1.49) per point of early-pregnancy E-DII. That estimate came from an observational dietary analysis within a trial, not a randomized comparison of DII reduction (20). A more recent Iranian prospective cohort reported a highest-versus-lowest quartile hazard ratio of 2.72 (1.11 to 6.63), with substantial uncertainty (21). Direct numerical comparison is limited because our estimate used one within-sample SD of ordinary DII, whereas the cited studies used tertiles, quartiles, or individual E-DII points and reported different effect measures.

Retrospective studies also require separate interpretation. In an Iranian case-control study with 122 cases and 266 controls, the highest DII tertile was associated with greater odds of GDM (OR 2.10, 95% CI 1.02 to 4.34), but the per-unit estimate was not statistically significant (OR 1.20, 0.94 to 1.54) (22). Another Iranian case-control study reported lower odds in the lowest than in the highest DII tertile (OR 0.52, 0.27 to 0.98) (23). A recalled prepregnancy dietary period does not make a questionnaire administered after case status was established a prospective exposure measurement. Recall and selection processes may therefore distinguish case-control estimates from those of prospective cohorts.

Important contrary findings prevent a simple positive-consensus interpretation. In Project Viva, higher pregnancy DII was associated with higher C-reactive protein but lower GDM odds (per-unit OR 0.78, 95% CI 0.65 to 0.95) (8). Soltani and colleagues found no highest-versus-lowest quartile association in 812 Iranian women (reported relative risk 0.97, 0.66 to 1.41) (9). The large nuMoM2b analysis found an association with a composite adverse pregnancy outcome, but its secondary GDM result was not statistically significant; the composite estimate should not be interpreted as a GDM estimate (24). In the Japan Environment and Children’s Study, results varied by BMI, and the published quartile labels ran from the most proinflammatory to the most anti-inflammatory diet. In the BMI 23 to below 25 kg/m² subgroup, the highest-numbered, most anti-inflammatory quartile had higher GDM odds than the most proinflammatory quartile. This was not evidence of a uniformly harmful association with greater dietary inflammatory potential (25).

Methodological differences offer plausible, although unproven, explanations for heterogeneity. Project Viva used first- and second-trimester FFQs, the Iranian prospective study assessed intake at 8 to 16 weeks, and the Finnish analysis used an early-pregnancy three-day food diary (8, 9, 20). Our FFQ was completed near OGTT and recalled the preceding three months. Scores also differed in parameter availability and energy handling. GDM ascertainment was not identical: the Iranian null cohort used fasting glucose and a 50-g one-hour test framework that differed from the diagnostic 75-g IADPSG OGTT used here (9). Population diet, prepregnancy adiposity, socioeconomic factors, and inclusion of contemporaneous weight gain in adjustment could further change the comparison. None of the available studies isolates the contribution of each methodological difference.

Evidence syntheses reflect that inconsistency. The 2023 review found conflicting results and did not support an overall association (10). Liu and colleagues subsequently reported a pooled OR of 1.17 (95% CI 1.01 to 1.36), with I² of 70%, and Hong and colleagues reported a per-unit pooled OR of 1.13 (1.02 to 1.25), with I² of 68.4% (11, 26). The positive pooled estimates were modest and heterogeneous. Reviews reuse many of the same primary cohorts, so their conclusions are not independent replications. Pooling observational studies also does not resolve exposure-timing problems or establish causality.

Empirically derived dietary patterns broaden the context but should not be substituted for DII evidence. A Nurses’ Health Study II analysis associated higher prepregnancy empirical dietary inflammatory pattern and empirical dietary index for hyperinsulinemia scores with GDM, and the Hefei cohort reported associations using the same families of indices (12, 27). Unlike the literature-derived DII, empirical inflammatory indices were developed from food-group patterns related to measured biomarkers (13). Agreement across different constructs may motivate further research on overall diet, but it does not validate the present 26-parameter score or establish that inflammation explains its association.

### 4.3 Measurement and interpretation of the association

The restricted parameter set is central to interpretation. Pregnancy studies have used, for example, 28 DII parameters in Project Viva, 29 in the Iranian prospective cohort, and 27 E-DII parameters in the NEST cohort (8, 9, 28). Such precedents show that incomplete dietary coverage is common; they do not demonstrate equivalence to the original 45-parameter DII or validate our particular subset. Excluding unavailable or unit-incompatible parameters avoids unsupported calculations but changes exposure coverage. The score may miss relevant foods and bioactive compounds, and its absolute distribution should not be compared uncritically across instruments or populations.

Energy handling also changes what is being measured. Energy contributed to the ordinary DII itself, and many absolute nutrient intakes varied with total intake. The additional energy-covariate model gave an estimate close to the primary result, suggesting that this particular adjustment did not remove the observed association. It did not demonstrate equivalence to E-DII or rule out energy-related measurement error. E-DII uses an energy-adjusted comparative database, whereas a regression covariate conditions on total energy after ordinary DII construction (15, 18).

Dietary assessment uncertainty was not limited to the DII algorithm. A validation study of a 61-item FFQ in Chinese pregnant women and the BIGCS food-frequency validation study provide useful methodological precedents, but neither establishes validity of the exact 103-item questionnaire used here (29, 30). The absence of an independently reproducible FFQ-to-nutrient conversion further limits confidence in absolute intake estimates. Reproducing DII from recorded nutrient values confirms the downstream calculation, not the accuracy of the underlying dietary measurement. A future validation study would need to evaluate the exact questionnaire, portion-size conversion, nutrient estimates, and resulting score in the intended pregnancy population.

The contrast between the continuous and quartile analyses should remain visible. Categorization can discard information, although that general principle cannot establish why our estimates differed (31). The quartile and continuous models also impose different exposure representations. Here, the quartile confidence intervals included potentially meaningful positive associations as well as the null, and the nonsignificant trend did not confirm a graded relationship. Similarly, a nonlinear P value of 0.888 indicates insufficient evidence against the modeled linear form, not proof that the relationship is exactly linear. Statistical significance in one representation and its absence in another should not be treated as a contradiction that can be resolved by selecting the more favorable result (19).

Questionnaire timing deserves particular weight. Restricting assessment to one to three days before OGTT reduced the estimate to 1.20 and widened its interval to include the null. That result remained compatible with the primary estimate, and no formal evidence established that timing caused the difference. Nevertheless, the restriction did not provide unequivocal confirmation. A longitudinal Chinese study documented dietary changes after GDM diagnosis, making reverse causation a credible concern for assessments close to diagnosis (32). Earlier clinical advice or suspicion could also affect recalled intake. The direction and magnitude of resulting bias in our study cannot be determined from the available timing data.

### 4.4 Biological and clinical context

An inflammatory pathway is biologically plausible but was not tested here. Pregnancy-related insulin resistance and beta-cell dysfunction interact with inflammatory processes, but a dietary score does not directly measure circulating inflammation (2). Pregnancy biomarker findings have themselves been mixed: Project Viva reported a positive DII–C-reactive protein association, a Tianjin study found timing-dependent and nonlinear associations with selected cytokines, and the NEST study did not support an association between E-DII and its measured circulating cytokines (8, 28, 33). The ROLO study related pregnancy E-DII to selected cardiometabolic measures, providing additional metabolic context without establishing DII as a GDM diagnostic test (34). We measured neither inflammatory mediators nor insulin resistance and therefore could not evaluate mediation.

Association also needs to be separated from prediction. In a prospective early-pregnancy biomarker study, individual biomarker areas under the receiver operating characteristic curve ranged from 0.466 to 0.573, indicating limited standalone discrimination in that sample (35). This does not show that dietary assessment is superior to biochemical testing. Our cross-sectional models were not developed or externally validated for prediction, and no evidence supports a DII screening threshold, incremental discrimination, calibration, or clinical net benefit from the present analysis.

Nutritional counseling should continue to follow established pregnancy guidance rather than the numerical DII cut points of this sample. Chinese guidance addresses clinical nutrition within the management of hyperglycemia in pregnancy (36). Broader trial evidence is informative but not uniform: the ESTEEM trial reported a reduction in the GDM component outcome with a Mediterranean-style intervention, while its primary maternal and offspring composite outcomes were not significantly reduced (37). UPBEAT did not reduce GDM despite improvements in some dietary and activity measures (38). In the PEARS secondary analysis, an intervention changed E-DII, but the reported inflammatory-score categories were not associated with GDM (39). None of those findings proves that lowering this study’s DII prevents GDM. If an inflammatory-diet concept is discussed in counseling, it should be used as an explanatory aid to an individualized dietary assessment, not a diagnostic label or treatment target. Food availability and socioeconomic circumstances matter, as illustrated by regional Chinese evidence linking socioeconomic position with pregnancy diet quality (40).

### 4.5 Strengths and limitations

The study combined records from three hospitals, applied the same explicit glucose thresholds, reported all included DII parameters and constants, and examined continuous, quartile, and nonlinear representations. Covariates were coded according to their measurement scale, hospital was included explicitly, and sensitivity analyses addressed energy, gestational weight gain, duplicate handling, and questionnaire timing. Independent recalculation supported the reproducibility of the downstream numerical analysis.

Several limitations constrain the contribution. The cross-sectional design and overlapping recall window prevented temporal and causal inference. Recruitment from tertiary hospitals in one city, without the eligible-attendee denominator or documented invitation mechanism, limited assessment of selection bias and generalizability to primary or secondary care and other regions. Exact-record matching was not equivalent to source-adjudicated participant identity. FFQ validity, simplified physical-activity measurement, upstream nutrient derivation, and laboratory comparability were incompletely documented. The 26-parameter score lacked local biomarker validation, and residual confounding could remain from supplement use, other pregnancy complications, unmeasured behaviors, or imperfect socioeconomic and activity measures. Prior regional evidence on diet quality cannot establish adequate control of socioeconomic confounding in Suzhou (40).

The absence of missing analytical values did not establish error-free source measurement. Sparse smoking data introduced a model concern despite a similar estimate after category combination. Confidence intervals were wide in several secondary analyses, and subgroup results did not establish interaction. No dated plan preceding outcome analysis was available, so the analytical hierarchy could not be described as prospectively registered. Finally, with GDM present in 21.0% of the analytical sample, the reported OR should not be read as a risk ratio: an OR of 1.26 does not mean a 26% increase in absolute probability or incidence.

## 5 Conclusion

A higher continuous 26-parameter DII was associated with higher adjusted odds of GDM among women attending three tertiary hospitals in Suzhou. Imprecise quartile estimates, a nonsignificant trend, and attenuation in timing-restricted and extreme-score-exclusion analyses limited the consistency of the findings. The study did not establish causality, a clinical threshold, or predictive utility. Prospective studies with dietary measurement clearly preceding metabolic assessment, validated and reproducible dietary instruments, and well-documented laboratory procedures are needed. Randomized dietary trials would be required to establish whether an intervention that changes dietary inflammatory potential reduces GDM incidence.

## Data Availability

The raw data supporting the conclusions of this article will be made available by the authors, without undue reservation.

## Supplementary material

Supplementary Table 1 Parameters used in the 26-parameter Dietary Inflammatory Index. Note: Constants were taken from the primary DII development paper. Calcium and vitamin A were not included. DII, Dietary Inflammatory Index; MUFA, monounsaturated fatty acids; PUFA, polyunsaturated fatty acids; SD, standard deviation.

Supplementary Table 2 Missingness of primary-model variables. Note: Denominator: 1,071 primary analytical records. No imputation was performed. DII, Dietary Inflammatory Index; GDM, gestational diabetes mellitus; BMI, body mass index.

## References

[1] Wu X Tiemeier H Xu T . Trends in gestational diabetes prevalence in China from 1990 to 2024: a systematic review and meta-analysis. Rev Endocr Metab Disord. (2025) 26:1009–21. doi: 10.1007/s11154-025-09987-0 40663307

[2] Plows JF Stanley JL Baker PN Reynolds CM Vickers MH . The pathophysiology of gestational diabetes mellitus. Int J Mol Sci. (2018) 19:3342. doi: 10.3390/ijms19113342 30373146 PMC6274679

[3] Tobias DK Zhang C Chavarro J Bowers K Rich-Edwards J Rosner B . Prepregnancy adherence to dietary patterns and lower risk of gestational diabetes mellitus. Am J Clin Nutr. (2012) 96:289–95. doi: 10.3945/ajcn.111.028266 22760563 PMC3396443

[4] Schoenaker DAJM Soedamah-Muthu SS Callaway LK Mishra GD . Pre-pregnancy dietary patterns and risk of gestational diabetes mellitus: results from an Australian population-based prospective cohort study. Diabetologia. (2015) 58:2726–35. doi: 10.1007/s00125-015-3742-1 26358582

[5] Shivappa N Steck SE Hurley TG Hussey JR Hébert JR . Designing and developing a literature-derived, population-based dietary inflammatory index. Public Health Nutr. (2014) 17:1689–96. doi: 10.1017/s1368980013002115 23941862 PMC3925198

[6] Shivappa N Steck SE Hurley TG Hussey JR Ma Y Ockene IS . A population-based dietary inflammatory index predicts levels of C-reactive protein in the Seasonal Variation of Blood Cholesterol Study (SEASONS). Public Health Nutr. (2014) 17:1825–33. doi: 10.1017/s1368980013002565 24107546 PMC3983179

[7] Zhang Z Wu Y Zhong C Zhou X Liu C Li Q . Association between dietary inflammatory index and gestational diabetes mellitus risk in a prospective birth cohort study. Nutr (Burbank Los Angeles County Calif). (2021) 87-88:111193. doi: 10.1016/j.nut.2021.111193 33774421

[8] Sen S Rifas-Shiman SL Shivappa N Wirth MD Hébert JR Gold DR . Dietary inflammatory potential during pregnancy is associated with lower fetal growth and breastfeeding failure: results from Project Viva. J Nutr. (2016) 146:728–36. doi: 10.3945/jn.115.225581 26936137 PMC4807648

[9] Soltani S Aminianfar A Hajianfar H Azadbakht L Shahshahan Z Esmaillzadeh A . Association between dietary inflammatory potential and risk of developing gestational diabetes: a prospective cohort study. Nutr J. (2021) 20:48. doi: 10.1186/s12937-021-00705-5 34078385 PMC8173880

[10] Bakhshimoghaddam F Razmi H Malihi R Mansoori A Ahangarpour A . The association between the dietary inflammatory index and gestational diabetes mellitus: a systematic review of observational studies. Clin Nutr ESPEN. (2023) 57:606–12. doi: 10.1016/j.clnesp.2023.08.007 37739712

[11] Liu RL Chen XQ Zheng QX Li JN Zhu Y Huang L . Impact of dietary inflammatory index on gestational diabetes mellitus in normal and overweight women: a systematic review and meta-analysis of observational studies. Asia Pacific J Clin Nutr. (2024) 33:298–312. doi: 10.6133/apjcn.202409_33(3).0002 38965719 PMC11389818

[12] Zu P Wang H Zhang L Bian H Bian J Jiang N . Insulinemic and inflammatory dietary patterns show enhanced predictive potential for gestational diabetes mellitus risk. J Clin Endocrinol Metab. (2025) 110:e741–9. doi: 10.1210/clinem/dgae238 38609168

[13] Tabung FK Smith-Warner SA Chavarro JE Wu K Fuchs CS Hu FB . Development and validation of an empirical dietary inflammatory index. J Nutr. (2016) 146:1560–70. doi: 10.3945/jn.115.228718 27358416 PMC4958288

[14] Lachat C Hawwash D Ocké MC Berg C Forsum E Hörnell A . Strengthening the reporting of observational studies in epidemiology-nutritional epidemiology (STROBE-nut): an extension of the STROBE statement. PloS Med. (2016) 13:e1002036. doi: 10.1371/journal.pmed.1002036 27270749 PMC4896435

[15] Hébert JR Shivappa N Wirth MD Hussey JR Hurley TG . Perspective: The dietary inflammatory index (DII)-lessons learned, improvements made, and future directions. Adv Nutr (Bethesda Md). (2019) 10:185–95. doi: 10.1093/advances/nmy071 30615051 PMC6416047

[16] Metzger BE Gabbe SG Persson B Buchanan TA Catalano PA International Association of Diabetes and Pregnancy Study Groups Consensus Panel . International association of diabetes and pregnancy study groups recommendations on the diagnosis and classification of hyperglycemia in pregnancy. Diabetes Care. (2010) 33:676–82. doi: 10.2337/dc09-1848 20190296 PMC2827530

[17] VanderWeele TJ . Principles of confounder selection. Eur J Epidemiol. (2019) 34:211–9. doi: 10.1007/s10654-019-00494-6 30840181 PMC6447501

[18] Willett WC Howe GR Kushi LH . Adjustment for total energy intake in epidemiologic studies. Am J Clin Nutr. (1997) 65:1220S–8S. doi: 10.1093/ajcn/65.4.1220s 9094926

[19] Greenland S Senn SJ Rothman KJ Carlin JB Poole C Goodman SN . Statistical tests, P values, confidence intervals, and power: a guide to misinterpretations. Eur J Epidemiol. (2016) 31:337–50. doi: 10.1007/s10654-016-0149-3 27209009 PMC4877414

[20] Pajunen L Korkalo L Koivuniemi E Houttu N Pellonperä O Mokkala K . A healthy dietary pattern with a low inflammatory potential reduces the risk of gestational diabetes mellitus. Eur J Nutr. (2022) 61:1477–90. doi: 10.1007/s00394-021-02749-z 34846602 PMC8921111

[21] Payandeh N Shahinfar H Jayedi A Mirmohammadkhani M Emadi A Shab-Bidar S . The association between the dietary inflammatory index during pregnancy and risk of gestational diabetes: a prospective cohort study and a meta-analysis. BMC Endocr Disord. (2025) 25:29. doi: 10.1186/s12902-025-01852-0 39901152 PMC11789316

[22] Shivappa N Hébert JR Akhoundan M Mirmiran P Rashidkhani B . Association between inflammatory potential of diet and odds of gestational diabetes mellitus among Iranian women. J Maternal-Fetal Neonatal Med. (2019) 32:3552–8. doi: 10.1080/14767058.2018.1466275 29661051 PMC6424648

[23] Moarefian A Kazeminejad S Gharang M Mansoori A . Adherence to the dietary inflammatory index and dietary approaches to stop hypertension and risk of gestational diabetes: a case-control study. BMC Pregnancy Childbirth. (2025) 25:987. doi: 10.1186/s12884-025-08123-9 41034786 PMC12487154

[24] Bharmal U Hayasaka M Saade G Kawakita T . Association between the dietary inflammatory index and adverse pregnancy outcomes. Am J Obstetrics Gynecology MFM. (2025) 7:101609. doi: 10.1016/j.ajogmf.2025.101609 39814139

[25] Kyozuka H Murata T Isogami H Imaizumi K Fukuda T Yamaguchi A . Preconception dietary inflammatory index and risk of gestational diabetes mellitus based on maternal body mass index: findings from a Japanese birth cohort study. Nutrients. (2022) 14:4100. doi: 10.3390/nu14194100 36235751 PMC9573395

[26] Hong L Zhu L Zhang J Fu Y Qi X Zhao M . Association of dietary inflammatory index with risk of gestational diabetes mellitus and preeclampsia: a systematic review and meta-analysis. Br J Nutr. (2024) 131:54–62. doi: 10.1017/s0007114523001678 37519248

[27] Liu P Yang J Tobias DK Tabung FK Hu FB Zhang C . Pre-pregnancy inflammatory and insulinemic dietary patterns and risk of gestational diabetes mellitus: a prospective cohort study of 14,674 women. BMC Med. (2025) 23:383. doi: 10.1186/s12916-025-04188-7 40597404 PMC12220632

[28] McCullough LE Miller EE Calderwood LE Shivappa N Steck SE Forman MR . Maternal inflammatory diet and adverse pregnancy outcomes: Circulating cytokines and genomic imprinting as potential regulators? Epigenetics. (2017) 12:688–97. doi: 10.1080/15592294.2017.1347241 28678596 PMC5687326

[29] Zhang H Qiu X Zhong C Zhang K Xiao M Yi N . Reproducibility and relative validity of a semi-quantitative food frequency questionnaire for Chinese pregnant women. Nutr J. (2015) 14:56. doi: 10.1186/s12937-015-0044-x 26040544 PMC4457005

[30] Yuan MY He JR Chen NN Lu JH Shen SY Xiao WQ . Validity and reproducibility of a dietary questionnaire for consumption frequencies of foods during pregnancy in the Born in Guangzhou Cohort Study (BIGCS). Nutrients. (2016) 8:454. doi: 10.3390/nu8080454 27483304 PMC4997369

[31] Altman DG Royston P . The cost of dichotomising continuous variables. BMJ (Clinical Res Ed). (2006) 332:1080. doi: 10.1136/bmj.332.7549.1080 16675816 PMC1458573

[32] Liu Y Guo N Dai Y Zhang L Li J Li X . A prospective observational study on maternal diet pre- and post-GDM diagnosis and pregnancy outcomes in individuals with/without GDM. BMC Pregnancy Childbirth. (2024) 24:754. doi: 10.1186/s12884-024-06961-7 39543524 PMC11566912

[33] Cui T Zhang J Liu L Xiong W Su Y Han Y . Relationship between the dietary inflammatory index score and cytokine levels in Chinese pregnant women during the second and third trimesters. Nutrients. (2022) 15:194. doi: 10.3390/nu15010194 36615851 PMC9824482

[34] Gainfort A Delahunt A Killeen SL O'Reilly SL Hébert JR Shivappa N . Energy-adjusted dietary inflammatory index in pregnancy and maternal cardiometabolic health: findings from the ROLO study. AJOG Global Rep. (2023) 3:100214. doi: 10.1016/j.xagr.2023.100214 37234814 PMC10205536

[35] Tigli A Baykus Y Deniz R Tari Selcuk K Sener N Degirmenci YE . Metabolic biomarkers in early detection of gestational diabetes mellitus: a prospective diagnostic accuracy study. Front Med. (2026) 13:1765602. doi: 10.3389/fmed.2026.1765602 41626233 PMC12852450

[36] Wang C Juan J Yang H Maternal-Fetal Medicine Committee, Chinese Society of Obstetrics and Gynecology, Chinese Medical Association Chinese Society of Perinatal Medicine, Chinese Medical Association Professional Committee of Gestational Diabetes Mellitus, Chinese Maternal and Child Health Association . A summary of Chinese guidelines on diagnosis and management of hyperglycemia in pregnancy (2022). Maternal-Fetal Med (Wolters Kluwer Health Inc). (2023) 5:4–8. doi: 10.1097/fm9.0000000000000181 40433235 PMC12106219

[37] H Al Wattar B Dodds J Placzek A Beresford L Spyreli E Moore A . Mediterranean-style diet in pregnant women with metabolic risk factors (ESTEEM): a pragmatic multicentre randomised trial. PloS Med. (2019) 16:e1002857. doi: 10.1371/journal.pmed.1002857 31335871 PMC6650045

[38] Poston L Bell R Croker H Flynn AC Godfrey KM Goff L . Effect of a behavioural intervention in obese pregnant women (the UPBEAT study): a multicentre, randomised controlled trial. Lancet Diabetes Endocrinol. (2015) 3:767–77. doi: 10.1016/s2213-8587(15)00227-2 26165396

[39] Killeen SL Phillips CM Delahunt A Yelverton CA Shivappa N Hébert JR . Effect of an antenatal lifestyle intervention on dietary inflammatory index and its associations with maternal and fetal outcomes: a secondary analysis of the PEARS trial. Nutrients. (2021) 13:2798. doi: 10.3390/nu13082798 34444958 PMC8399423

[40] Li S Lei F Zhang R Liu D Qu P Cheng Y . Socioeconomic disparity in the diet quality of pregnant women in Northwest China. Asia Pacific J Clin Nutr. (2019) 28:330–40. doi: 10.6133/apjcn.201906_28(2).0015 31192562

