# Supplementary material for: Dietary inflammatory index and gestational diabetes mellitus in a multicenter cross sectional study in Suzhou
Source: Front Immunol. 2026 Oct 5;17:1909211. doi: 10.3389/fimmu.2026.1909211 (PMC13677459; doi:10.3389/fimmu.2026.1909211)
Supplement: Supplementary file 1 [file Table1.docx]

**Supplementary material**

**Supplementary Table S1. Parameters used in the 26-parameter Dietary Inflammatory Index**

| **Parameter** | **Unit** | **Global mean** | **Global SD** | **Inflammatory effect score** |
| --- | --- | --- | --- | --- |
| energy | kcal/day | 2056 | 338 | 0.180 |
| carbohydrate | g/day | 272.2 | 40 | 0.097 |
| protein | g/day | 79.4 | 13.9 | 0.021 |
| total fat | g/day | 71.4 | 19.4 | 0.298 |
| fiber | g/day | 18.8 | 4.9 | -0.663 |
| cholesterol | mg/day | 279.4 | 51.2 | 0.110 |
| saturated fat | g/day | 28.6 | 8 | 0.373 |
| MUFA | g/day | 27 | 6.1 | -0.009 |
| PUFA | g/day | 13.88 | 3.76 | -0.337 |
| omega 3 | g/day | 1.06 | 1.06 | -0.436 |
| omega 6 | g/day | 10.8 | 7.5 | -0.159 |
| beta carotene | microgram/day | 3718 | 1720 | -0.584 |
| vitamin C | mg/day | 118.2 | 43.46 | -0.424 |
| vitamin D | microgram/day | 6.26 | 2.21 | -0.446 |
| vitamin E | mg/day | 8.73 | 1.49 | -0.419 |
| thiamin | mg/day | 1.7 | 0.66 | -0.098 |
| riboflavin | mg/day | 1.7 | 0.79 | -0.068 |
| niacin | mg/day | 25.9 | 11.77 | -0.246 |
| vitamin B6 | mg/day | 1.47 | 0.74 | -0.365 |
| vitamin B12 | microgram/day | 5.15 | 2.7 | 0.106 |
| folic acid | microgram/day | 273 | 70.7 | -0.190 |
| iron | mg/day | 13.35 | 3.71 | 0.032 |
| magnesium | mg/day | 310.1 | 139.4 | -0.484 |
| zinc | mg/day | 9.84 | 2.19 | -0.313 |
| selenium | microgram/day | 67 | 25.1 | -0.191 |
| alcohol | g/day | 13.98 | 3.72 | -0.278 |

*Note: Constants were taken from the primary DII development paper. Calcium and vitamin A were not included. DII, Dietary Inflammatory Index; MUFA, monounsaturated fatty acids; PUFA, polyunsaturated fatty acids; SD, standard deviation.*

**Supplementary Table S2. Missingness of primary-model variables**

| **Variable** | **Missing count** | **Missing percentage** |
| --- | --- | --- |
| Hospital | 0 | 0.0% |
| Age | 0 | 0.0% |
| Prepregnancy BMI | 0 | 0.0% |
| Education | 0 | 0.0% |
| Household income | 0 | 0.0% |
| Parity | 0 | 0.0% |
| Gestational week | 0 | 0.0% |
| Physical activity | 0 | 0.0% |
| Smoking | 0 | 0.0% |
| Secondhand smoke | 0 | 0.0% |
| 26-parameter DII | 0 | 0.0% |
| GDM | 0 | 0.0% |
| Family history of type 2 diabetes | 0 | 0.0% |
| Previous GDM | 0 | 0.0% |

*Note: Denominator: 1,071 primary analytical records. No imputation was performed. DII, Dietary Inflammatory Index; GDM, gestational diabetes mellitus; BMI, body mass index.*
